# The Pain–to–Well-Being Relationship in Patients Experiencing Chronic Orofacial Pain

**DOI:** 10.3389/fneur.2020.557415

**Published:** 2020-12-03

**Authors:** Kanokporn Bhalang, Beat Steiger, Nenad Lukic, Aleksandra Zumbrunn Wojczyńska, Ray S. Hovijitra, Dominik A. Ettlin

**Affiliations:** ^1^Department of Oral Medicine, Faculty of Dentistry, Chulalongkorn University, Bangkok, Thailand; ^2^Department of Psychiatry and Psychotherapy, Psychiatric Services Aargau, Aarau, Switzerland; ^3^Orofacial Pain Unit, Center of Dental Medicine, University of Zurich, Zurich, Switzerland; ^4^Dental Center, Bumrungrad International Hospital, Bangkok, Thailand; ^5^São Leopoldo Mandic Institute and Research Center, São Paulo, Brazil; ^6^Department of Reconstructive Dentistry and Gerodontology, School of Dental Medicine, University of Berne, Berne, Switzerland

**Keywords:** injustice, stress, well-being, orofacial pain, dysmorphic

## Abstract

**Introduction:** Orofacial pain features may negatively influence a person's well-being and vice versa. Some aspects of well-being can be measured with axis II instruments that assess patients' psychosocial and behavioral status. The aim of this study was to investigate associations between pain features and psychosocial variables as indicators of well-being.

**Materials and Methods:** Seven hundred ninety-nine anonymized datasets collected using the Web-based Interdisciplinary Symptom Evaluation (WISE) of patients reporting to the Interdisciplinary Orofacial Pain Unit, University of Zurich, between March 19, 2017 and May 19, 2019, were analyzed. Pain features including intensity, number of locations, impact, and duration were evaluated. Psychometric measures assessed pain-related catastrophizing and disability, illness perception, distress, anxiety, depression, injustice experience, dysmorphic concerns, and insomnia.

**Results:** Most patients were between 30 and 59 years old (58.3%), female (69.8%), working (66.0%), and experienced pain for more than 6 months (68.5%). Pain intensities were higher in women than men and higher in disabled than working patients. Scores indicating elevated stress and depression were also observed in disabled patients. The sample prevalence rates of clinically relevant axis II instrument scores were as follows: Graded Chronic Pain Scale for the Head (GCPS-H), 27%; Patient Health Questionnaire 4 (PHQ4), 21%; PHQ9, 21%; Pain Catastrophizing Scale (PCS), 20%; General Anxiety Disorder 7 (GAD7), 15%; Insomnia Severity Index (ISI), 15%; Injustice Experience Questionnaire (IEQ), 14%; GCPS for the Body (GCPS-B), 13%; PHQ for Stress (PHQstr), 6%; and Dysmorphic Concern Questionnaire (DCQ), 2%. Noteworthy results of correlation analysis of the clinically relevant axis II scores and pain measures were as follows: the PHQstr had moderate associations (0.34–0.43) with the sum of pain intensity at rest and during function, number of pain locations, and typical pain intensity. The IEQ scores were moderately associated with typical pain intensity at 0.39. The DCQ scores were moderately associated with pain extension at 0.41.

**Conclusions:** Moderate correlations of certain pain and well-being measures were found in patients reporting clinically relevant stress, injustice experience, and dysmorphic concern, all of which reflect impaired well-being. PHQ4 is suitable for routine distress screening in the clinical setting.

## Introduction

Pain is a complex, multidimensional construct that includes features such as intensity (at rest and during movements), spread, symmetry, chronicity, and impact of pain. Analogous to pain in any other body region, orofacial pain (OFP) negatively influences well-being and *vice versa* (1). Well-being can be conceptualized as a spectrum, with happiness, minimal distress, and high well-being at one end and elevated depression, anxiety, and low well-being at the other (2). Diverse psychometric instruments exist for indirectly measuring well-being, e.g., the Hospital Depression and Anxiety Scale, the General Health Questionnaire, the Perceived Stress Scale, and the Positive and Negative Affect Schedule (3). Accordingly, a well-being proxy measure may consist of a diverse set of psychometric instruments that offer information on a patient's psychological and behavioral status. From a pain management perspective, well-being proxy measures, such as anxiety, depression, catastrophizing, and others, are all relevant in influencing treatment outcomes in patients experiencing OFP and temporomandibular disorders (TMDs) (4–7). Another important negative influence on well-being is unemployment, resulting in feelings of worry, insecurity, and stress due to changes in the patient's financial situation (8, 9).

Pain and well-being are interrelated. Hence, a dual-axis diagnostic system has been proposed for evaluating patients experiencing OFP and TMD (10). This system differentiates physical diagnoses (which can be grouped into axis I) from the patients' psychosocial and behavioral status (known as axis II) reflecting their well-being. Notably, axis I diagnoses are categorical in nature, such as temporomandibular joint disc displacement or arthritis, while axis II construct measures have ordinal values. According to an international expert panel, axis II instruments aim at assessing the psychosocial and behavioral status (including pain-related disability) (11). For the purpose of this paper, we refer to pain as a symptom and not as a diagnosis or psychological status *per se*. Sleep has been proposed as an additional axis II construct because a strong relationship between self-assessed sleep disturbances, OFP, and TMD has been observed (12, 13). Perceived injustice, anger, illness perception, catastrophization, and dysmorphophobia are additional potential moderators of the pain–to–well-being relationship (14–17).

The Web-based Interdisciplinary Symptom Evaluation (WISE) is an online tool based on self-reports that assists clinicians in the comprehensive assessment of patients with OFP or TMD (18). WISE records sex, age, and employment status. This evaluation further combines a description of pain features with widely available in-depth psychometric measures. The automatically generated summary report supports clinicians in identifying case complexity and disability levels. WISE thus facilitates resource planning for the initial consultation, such as allocation of time and appropriate healthcare specialists. Anonymized WISE data can be extracted for research purposes.

Using anonymous data collected by the WISE tool, the aim of this study was to correlate pain features with other axis II construct measures reflecting well-being in patients experiencing OFP of mixed etiologies. Further, we analyzed whether known confounders such as sex, age, employment status, and pain duration influenced psychometric scores and pain features.

## Materials and Methods

### Subjects

This study included 799 anonymized WISE datasets of patients reporting to the Interdisciplinary Orofacial Pain Unit, Center of Dental Medicine, University of Zurich, Switzerland, between March 19, 2017 and May 19, 2019. The spectrum of axis I diagnoses encountered in this unit has been reported elsewhere (16). Patients completed the WISE questionnaire prior to their first clinical appointment. Anonymized data were retrieved from a server located at Hof University of Applied Science, Germany, and exported in csv format for statistical analysis. According to Swiss law, researchers can use strictly anonymized data and do not require approval by an ethics committee. Only fully completed WISE datasets of patients reporting OFP were analyzed, given that the subjects consented to the use of their anonymized data for research. Data related to non-painful symptom reports were excluded.

### Web-Based Interdisciplinary Symptom Evaluation Datasets

The WISE system is a Web-based instrument for interdisciplinary subject-tailored symptom evaluation in patients experiencing OFP and TMD (18). WISE combines a symptom-oriented checklist with validated in-depth questionnaires, also referred to as case finding instruments. The questionnaires are presented when the checklist scores exceed threshold values and thus indicate a burden related to the screening item. Graphical maps offer patients the opportunity to indicate their pain location, intensity, and duration in various defined oral and cranial tissues as well as other body regions. In addition to pain measures, several other psychometric instruments are integrated. Only German and English versions of the WISE were completed. German versions of the applied measures are available. The following variables from the WISE datasets were used in this study.

#### Pain Features

**Figure 1:**
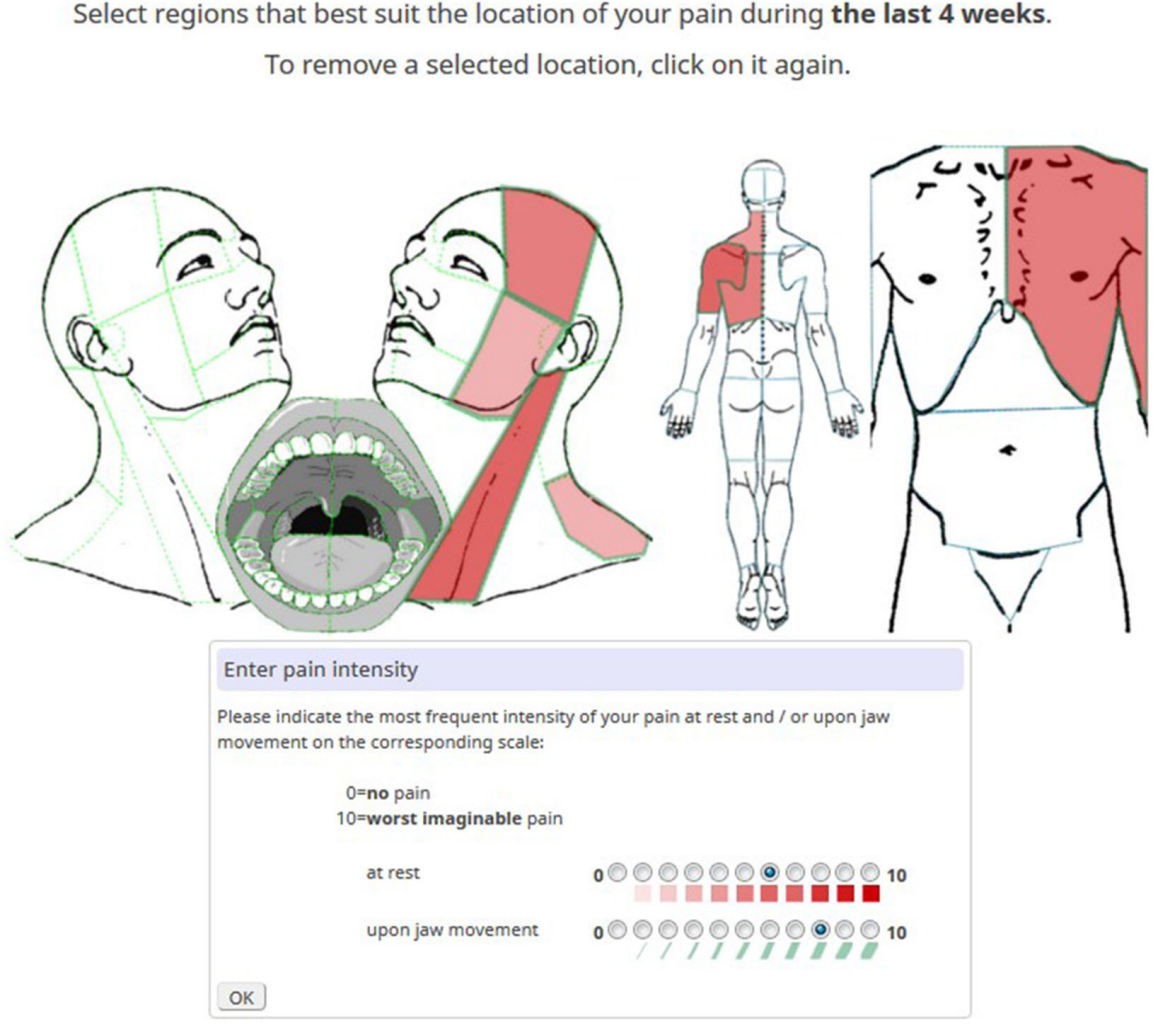
Example of a pain diagram with marked pain locations. The most intense red hue signifies the highest pain intensity in the sternocleidomastoid muscle. The broadest green border reflects the highest pain intensity upon jaw movement in the masseter area.

Pain features were captured by detailed interactive pain maps of the head and neck and front/back view maps of the rest of the body. Pain locations were differentiated by 93 selectable anatomical areas across the entire body and 72 areas across the head and neck region (Figure 1). Patients marked painful areas by clicking on a defined area and reported the typical pain intensity at rest and upon movement. Both were reported for the last 4 weeks on an 11-point numeric rating scale (NRS) for pain. Different pain intensities at rest were represented by gradients of red, and different pain intensities upon movement were captured by graded widths of anatomical green borders.

Using the same NRS, typical (PI-typ) and maximum (PI-max) pain intensities of the chief complaint were additionally reported. Pain extension for the body (P-ext-B) was defined as the total number of pain locations across the entire body and pain extension for the head (P-ext-H) was defined as the number of pain locations across the head and neck region (left part of Figure 1). Pain impact at rest and pain impact upon movement were calculated as the sum scores of pain intensities across the head and neck region at rest (∑-PI-rest-H) and upon movement (∑-PI-move-H), respectively. The duration of OFP (P-dur) as a measure of chronicity was grouped by intervals: <3 months, 3–6 months, 6 months−2 years, 2–5 years, >5 years.

#### Axis II Psychometric Measures

##### Brief Illness Perception Questionnaire

The Brief Illness Perception Questionnaire (B-IPQ) (19–21) assesses cognitive and emotional representations of illness and health threat. Eight questions covering different aspects of illness perception were rated on an NRS ranging from 0 to 10. The maximum score is 80. No cutoff score has been reported for this questionnaire. Test–retest reliability of the single items of the B-IPQ ranged from 0.48 to 0.70; a coefficient alpha value was not reported.

##### Dysmorphic Concern Questionnaire

The Dysmorphic Concern Questionnaire (DCQ) (22, 23) assesses excessive preoccupation with an imagined or a real minimal defect in appearance that is associated with a significant impact on psychosocial functioning. The questionnaire consists of seven items that cover different aspects of dysmorphic concern. Each item is rated on an ordinal scale ranging from 0 to 3, resulting in a maximum sum score of 21. A cutoff score of nine indicates a possible body dysmorphic disorder. The following coefficient alpha for the DCQ has been reported: 0.85; test–retest reliability was not reported.

##### Graded Chronic Pain Scale Version 2.0 for the Head or for the Body

The Graded Chronic Pain Scale (GCPS) (24) consists of three pain intensity scales ranging from “no pain” (=0) to “pain as bad as it could be” (=10) assessing current, worst, and average pain intensity for the last 30 days. The disability days report the number of days being kept from daily activities by pain for the last 3 months. The number of days determines a disability score ranging from 0 to 3. Three additional scales measure the interference by pain during (1) daily activities, (2) recreational, social, and family activities, and (3) working ability on a scale from “no interference” (=0) to “unable to carry on any activities” (=10) for the last 30 days. The average value of these three disability scales yields a disability score ranging from 0 to 3. Combining the pain activity interference score with the disability day score results in a disability sum score ranging from 0 to 6. Two separate disability scores due to pain in the head (GCPS-H) or the body (GCPS-B) were calculated. Scores ≥3 are considered as high disability and severely limiting. The following coefficient alpha has been reported: 0.71 for TMD pain; test–retest reliability has not been reported.

##### General Anxiety Disorder 7

The General Anxiety Disorder 7 (GAD7) (25) assesses general anxiety in primary care patients. Seven items cover different aspects of general anxiety. For the question “Over the last 2 weeks, how often have you been bothered by the following problems? “items are scored on a four-point scale from not at all” (=0), “several days” (=1), “half of the days” (=2), and “nearly every day” (=3). Summary scores range from 0 to 21 and indicate anxiety levels of “none/minimal” (0–4), “mild” (5–9), “moderate” (10–14), or “severe” (>14). The following coefficient alpha has been reported: 0.92; for test–retest reliability: 0.83.

##### Injustice Experience Questionnaire

The Injustice Experience Questionnaire (IEQ) (26–28) assesses injustice experienced due to accidents, injuries, or maltreatment. Twelve items reflect the frequency of thoughts, beliefs, and emotions associated with injury. They are rated on a scale with the following response options: “never” (=0), “rarely” (=1), “sometimes” (=2), “often” (=3), to “all the time” (=4). The maximum score is 48. Scores ≥18 indicate the need for professional evaluation. The following coefficient alpha has been reported: 0.92; for test–retest reliability: 0.90.

##### Insomnia Severity Index

The Insomnia Severity Index (ISI) (29, 30) screens for sleep disorders by measuring the severity of insomnia problems, sleep-related satisfaction, and interference on a scale of “none” (=0), “mild” (=1), “moderate” (=2), “severe” (=3), and “very severe” (=4) for the items falling asleep, staying asleep, and waking up too early over the past 2 weeks. On a scale from “very satisfied” (=0), “satisfied” (=1), “moderately satisfied” (=2), “dissatisfied” (=3), to “very dissatisfied” (=4), the patient indicates how satisfied/dissatisfied he/she perceives his/her current sleep pattern. On three additional scales from “not at all” (=0), “a little” (=1), “somewhat” (=2), “much” (=3), to “very much” (=4), the patient is asked how much he/she considers his/her sleep problem to interfere with daily activities, how noticeable the interference by his/her sleep problem is to others, and how worried/distressed the patient is because of his/her sleep problem. The maximum score is 28, with scales of “none” (0–7), “subthreshold” (8–14), “moderate” (15–21), or “severe” (>21). The following coefficient alpha has been reported: 0.74; test–retest reliability was not reported.

##### Pain Catastrophizing Scale

The Pain Catastrophizing Scale (PCS) (31, 32) assesses catastrophizing thoughts and corresponding behavior. The questionnaire has 13 items that are scored on a five-point ordinal scale [“not at all” (=0), “to a slight degree” (=1), “to a moderate degree” (=2), “to a great degree” (=3), or “all the time” (=4)]. Scores can also be calculated for three subscales of helplessness, maximizing, and ruminating. The maximum value is 52 for the entire questionnaire. The corresponding cutoff values are 13 for helplessness, five for maximizing, 13 for ruminating, and 30 for the entire questionnaire. These cutoff values correspond to the 75th percentile. The following coefficient alpha has been reported: 0.87; for test–retest reliability: 0.75.

##### Patient Health Questionnaire 4

The Patient Health Questionnaire 4 (PHQ4) (33) screens for anxiety and depression. This questionnaire consists of two subscales, GAD2 (items one and two of the GAD7) and PHQ2 (items one and two of the PHQ9). Items are scored on an ordinal scale ranging from 0 to 3 using the same labels as the GAD7. Scores can be calculated for the two subscales (maximum score = 6) and overall (maximum score = 12). Scores ≥6 for the total score and three for the sub-scores indicate expert evaluation referral. The following coefficient alpha has been reported: 0.87; for test–retest reliability: 0.81.

##### Patient Health Questionnaire 9

The PHQ9 (34, 35) assesses the severity of depression. Nine items covering different aspects of depression are scored on an ordinal scale ranging from 0 to 3 using the same labels as the GAD7. Summary scores range from 0 to 27, indicating depression levels of “none/minimal” (0–4), “mild” (5–9), “moderate” (10–14), “moderately severe” (15–19), or “severe” (>19). A cutoff score range of 8–11 has been recommended for expert evaluation referral. The following coefficient alpha has been reported: 0.89; for test–retest reliability: 0.84.

##### Patient Health Questionnaire for Stress

The Patient Health Questionnaire for Stress (PHQstr) (34, 36) is a 10-item subscale of the Primary Care Evaluation of Mental Disorders (PRIME-MD) Patient Health Questionnaire that addresses psychosocial stress burden. Items are scored on an ordinal scale ranging from “not at all” (=0), “a little” (=1), to “a lot” (=2). Summary scores range from 0 to 20 indicating the degree of psychosocial stress burden by the following levels: “none/minimal” (0–4), “mild” (5–9), “medium” (10–14), or “severe” (>14). No coefficient alpha nor test–retest reliability was reported.

### Statistical Analysis

The data were analyzed using SPSS (Version 22, Armonk, New York). Descriptive statistics were used to characterize the datasets. Spearman rank correlation was used for correlation analysis. To determine the effect size, small (0.1 < *r* < 0.3), moderate (0.3 < *r* < 0.5), and strong (*r* ≥ 0.5) were used for the different association levels (37). Analysis of variance was used with a Bonferroni or Tamhane correction for *post hoc* testing depending on whether the variances were equal or not. A significance level of ≤0.05 was considered statistically significant.

## Results

Most patients were female (*N* = 558; 69.8%), with a female-to-male ratio of 2.3:1. Among the patients, 73.6% (*N* = 588) were 20–59 years old, 66.0% were working (*N* = 527), and 84.4% suffered from pain for more than 3 months (*N* = 675) (Table 1).

**Table 1:** Sample characteristics (*N* = 799).

**Gender**		**Age group**							**Employment status**					**Pain duration**				
**F**	**M**	**10–19**	**20–29**	**30–39**	**40–49**	**50–59**	**60–69**	**≥70**	**Training**	**Working**	**Retired**	**Disabled**	**No job**	**<3 months**	**3–6 months**	**6 months−2 years**	**2–5 years**	**>5 years**
558	241	53	122	148	162	156	92	66	80	527	113	52	27	124	128	248	149	150
69.8%	30.2%	6.6%	15.3%	18.5%	20.3%	19.5%	11.5%	8.2%	10.0%	66.0%	14.1%	6.5%	3.4%	15.5%	16.0%	31.0%	18.6%	18.8%

The descriptive statistics of various pain measures are shown in Table 2. On average, patients had 8.69 pain sites across the entire body and 5.54 pain sites across the head region. The mean typical pain intensity was 4.91, and the mean maximum pain intensity was 7.10. With an average of 33.4, the intensity sum score at rest was lower compared with upon movement (38.2).

**Table 2:** Descriptive statistics of pain measures used in the study (*N* = 799).

**Pain measures**	**Maximum possible score**	**Mean**	**SD**
Number of pain locations across the entire body (P-ext-B)	93	8.69	9.56
Number of pain locations across the head and neck region (P-ext-H)	72	5.54	6.91
Typical pain intensity (PI-typ)	10	4.91	2.44
Maximum pain intensity (PI-max)	10	7.10	2.49
Pain intensity sum score at rest across the head and neck region (∑PI-rest-H)	720	33.4	48.7
Pain intensity sum score upon movement across the head and neck region (∑PI-move-H)	720	38.2	51.3

**Figure 2:**
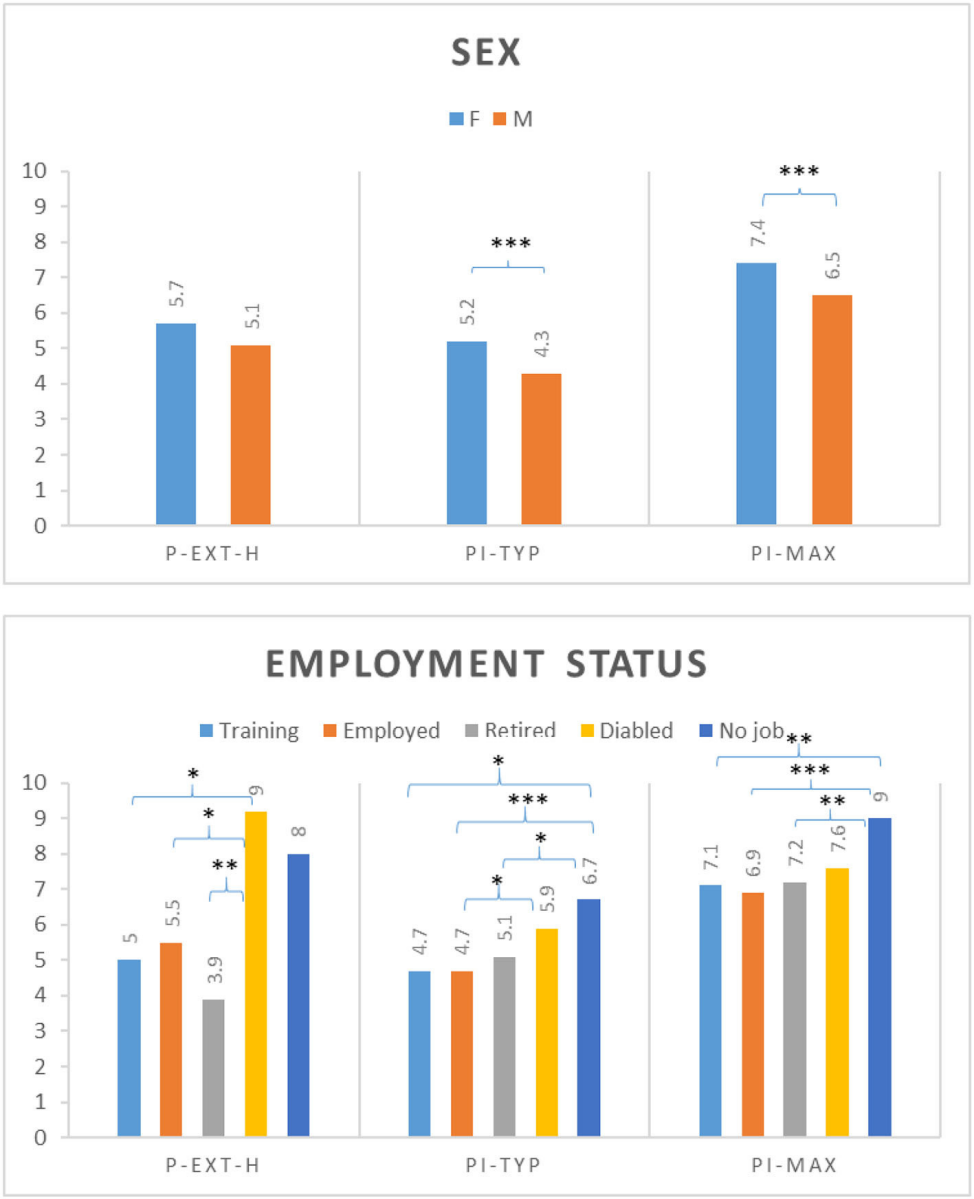
Analysis of variance between selected patient characteristics (sex, employment status) and selected pain features (P-ext-H, number of pain locations across the head and neck region; PI-typ, typical pain intensity; PI-max, maximum pain intensity); *Post hoc* tests with Bonferroni correction or Tamhane correction (*P ≤ 0.05 **P ≤ 0.01 ***P ≤ 0.001).

We determined the influence of patient characteristics (sex, age, employment status, and pain duration) on the mean of the assessed pain features. Sex had a significant influence on PI-typ, PI-max (Figure 2), and ∑-PI-rest-H, with women reporting higher mean values. Effects of age were found for P-ext; patients aged 30–39 years indicated more pain locations compared with older patients (>60 years old). Employment status demonstrated significant effects on all pain-related measures. Disabled patients significantly indicated more pain locations compared to employed patients, patients in training, or retired patients. Working patients presented more pain locations compared to retired patients. The highest typical and maximum pain intensities were reported by patients without a job and by disabled patients (Figure 2). The ∑-PI-rest-H was highest in disabled patients. Pain duration had significant effects on all pain-related measures, except for PI-typ and PI-max. The number of pain locations, ∑-PI-rest-H, and ∑-PI-move-H increased with the duration of pain.

The number of patients who completed one of the case finding questionnaires ranged from 216 (GAD7) to 799 (PCS, PHQ4, and PHQstr). The percentage of patients reaching a clinically relevant score (cutoff) ranged from 5.1% (DCQ) to 73.7% (PHQ9) (Table 3).

**Table 3:** Scores of the axis II psychometric measures [Note that ≥ cutoff score (CO) indicates clinical relevance].

**Domains (alphabetical order)**	***N***	**Max**	**Mean**	**SD**	**≥ cut-off score CO**			
					**CO**	***N***	**% domain**	**% of study sample**
Dysmorphic Concern Questionnaire (DCQ)	432	21/21	1.45	3.35	9	22	5.10	3
General Anxiety Disorder 7 (GAD7)	216	21/21	10.8	3.81	10	120	55.6	15
Graded Chronic Pain Scale-Head (GCPS-H)	688	6	1.79	0.84	3	218	31.7	27
Graded Chronic Pain Scale-Body (GCPS-B)	576	6	1.04	1.62	3	107	18.5	13
Illness Perception Questionnaire (IPQ)	566	78/80	44.5	11.5				
Injustice Experience Questionnaire (IEQ)	302	48/48	14.1	11.3	18	108	35.8	14
Insomnia Severity Index (ISI)	296	28/28	13.5	5.58	15	121	40.9	15
Pain Catastrophizing Scale (PCS)	799	51/52	16.6	12.6	30	157	19.6	20
Patient Health Questionnaire 4 (PHQ4)	799	12/12	3.13	3.10	6	165	21.0	21
Patient Health Questionnaire 9 (PHQ9)	232	25/27	12.5	4.81	10	171	73.7	21
Patient Health Questionnaire Stress (PHQstr)	799	20/20	6.24	3.74	10	46	18.9	6

**Figure 3:**
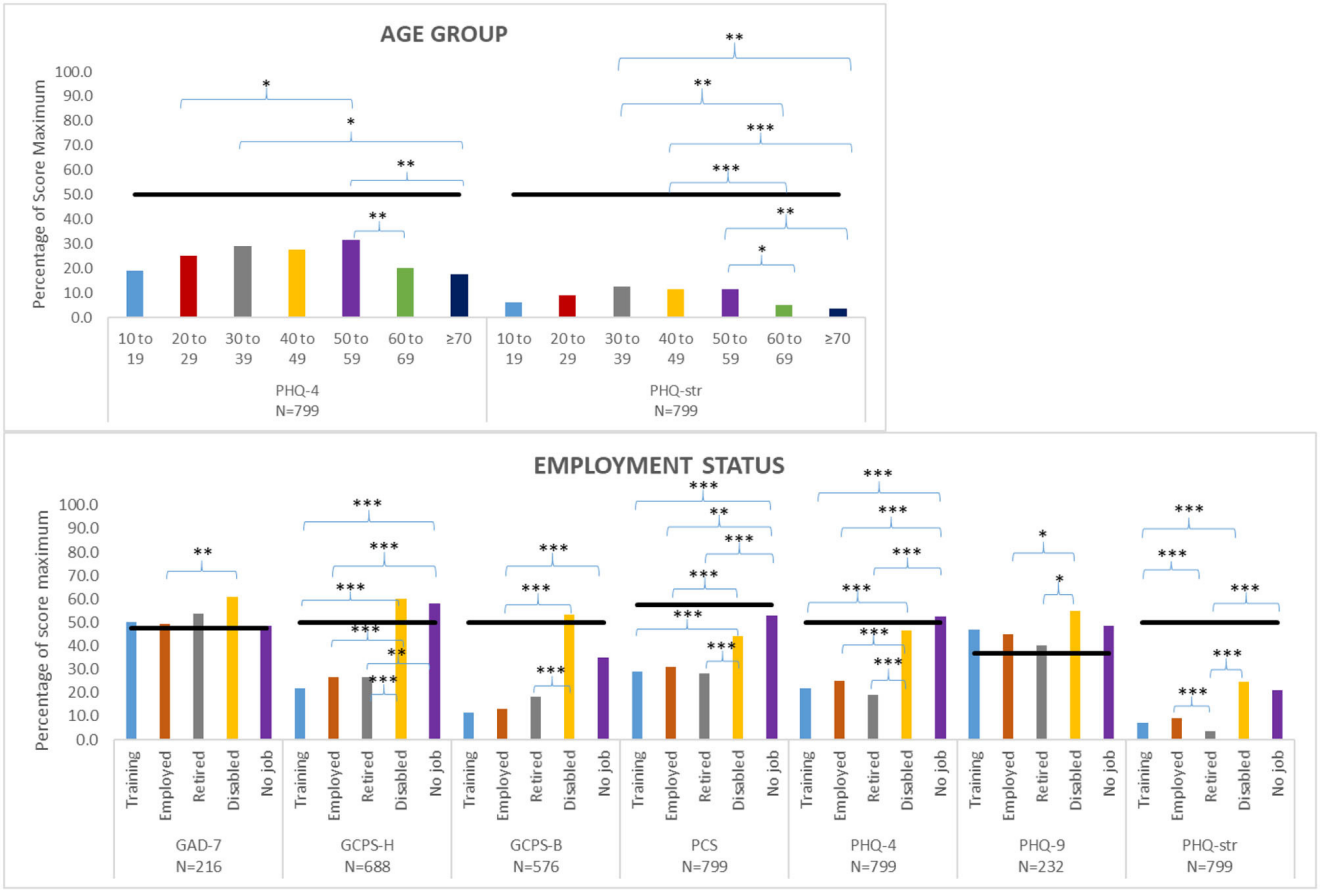
Analysis of variance between selected patient characteristics (age group, employment status) and selected axis II measures [Patient Health Questionnaire 4 (PHQ4), Patient Health Questionnaire Stress (PHQstr), General Anxiety Disorder 7 (GAD7), Graded Chronic Pain Scale-Head (GCPS-H), Graded Chronic Pain Scale-Body (GCPS-B), Pain Catastrophizing Scale (PCS), Patient Health Questionnaire 9 (PHQ9)]; *Post hoc* tests with Bonferroni correction or Tamhane correction (*P ≤ 0.05 **P ≤ 0.01 ***P ≤ 0.001). Maximum scores are listed in Table 3. Black bars indicated cutoff scores of the respective psychometric instrument.

The analysis of variance of the selected mean axis II domain scores grouped by selected patient characteristics (age group and employment status) is shown in Figure 3. There were no significant effects of age on axis II measures, except that older patients (>60 years) had lower PHQstr scores than those aged 30–59 years, and patients in the middle age groups had a higher PHQ4 score than those in the younger age groups and in older patients. Employment status had a significant effect on several measures. GCPS-H scores were higher among disabled patients or patients with no job than among trainees, working, or retired patients; GCPS-B scores were higher in disabled patients than in trainees, working, or retired patients. PCS and PHQ4 values were higher in disabled patients or patients with no job compared with trainees, working, or retired patients. PHQ9 scores were higher in disabled than in retired or working patients, and PHQstr scores were higher in disabled patients than in trainees, working, or retired patients. Pain duration had no significant effect on psychosocial variables except for PHQ4 and PHQstr, where patients experiencing pain longer than 5 years had higher mean values compared with patients with a pain duration of <3 months.

**Figure 4:**
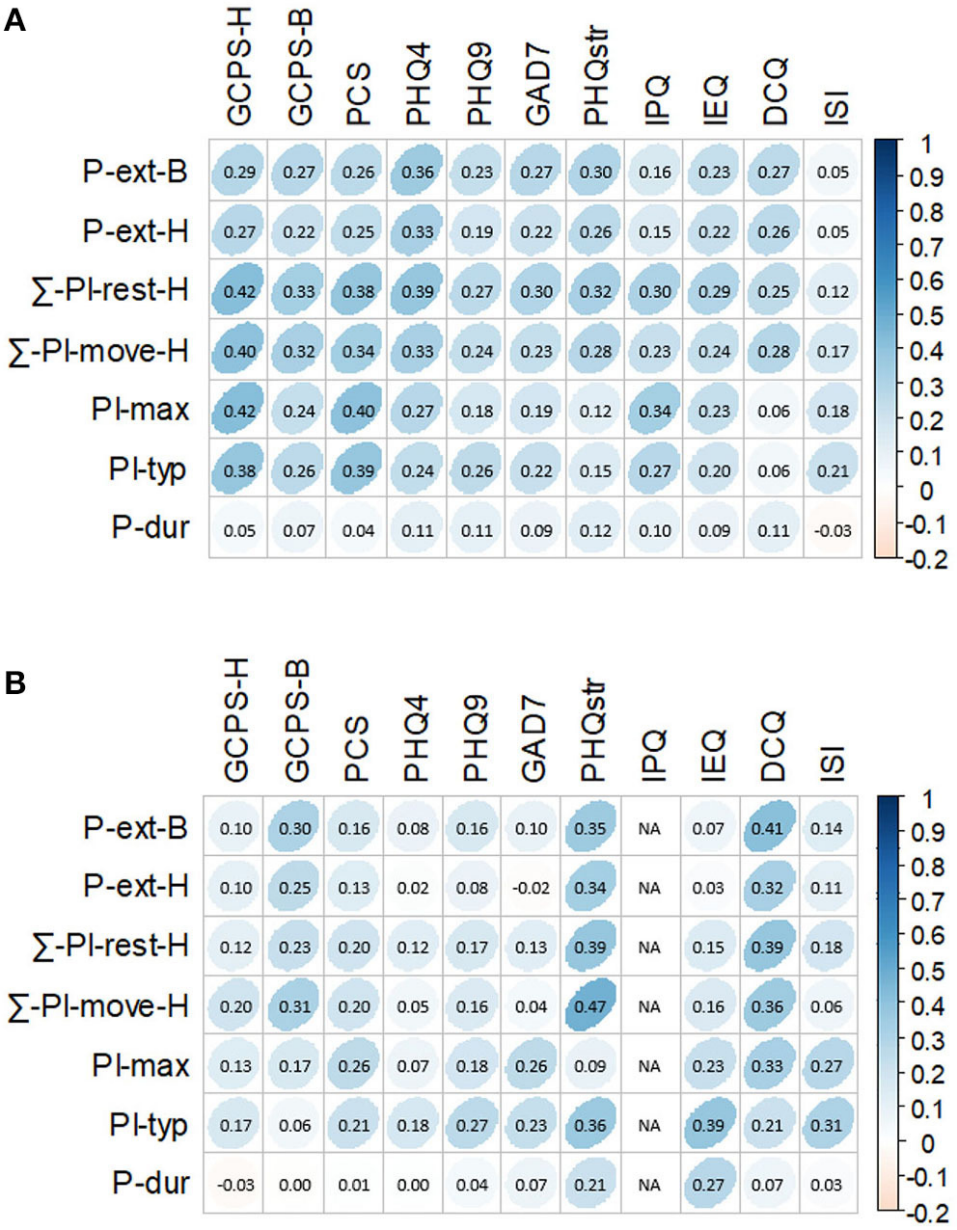
Correlations between scores of pain measures and scores of axis II measures as proxies for well-being [Pain measures number of pain locations across the entire body (P-ext-B), number of pain locations across the head and neck region (P-ext-H), summary score of pain intensity at rest across the head and neck region (∑-PI-res-H), summary score of pain intensity upon movement across the head and neck region (∑-PI-move-H), maximum pain intensity (PI-max), typical pain intensity (PI-typ), and pain duration (P-dur) and axis II measures Graded Chronic Pain Scale-Head (GCPS-H), Graded Chronic Pain Scale-Body (GCPS-B), Pain Catastrophizing Scale (PCS), Patient Health Questionnaire 4 (PHQ4), Patient Health Questionnaire 9 (PHQ9), General Anxiety Disorder 7 (GAD7), Patient Health Questionnaire Stress (PHQstr), Illness Perception Questionnaire (IPQ), Injustice Experience Questionnaire (IEQ), Dysmorphic Concern Questionnaire (DCQ), and Insomnia Severity Index (ISI); Spearman rank correlation was used for the analysis (*P* = 0.05), and darker blue shades represent higher correlations]. **(A)** Correlations between scores of pain measures and all scores of axis II measures. **(B)** Correlations between scores of pain measures and scores above clinical relevance of axis II measures.

For the correlations between various pain features and axis II measures (Figure 4A), the following five pain features showed moderate correlation effects (*r* ≥ 0.30; *P* < 0.50) with other axis II measures: (1) ∑-PI-rest-H with PCS, PHQ4, PHQstr, GAD7, IPQ, GCPS-H, and GCPS-B; (2) ∑-PI-move-H with PCS, PHQ4, GCPS-H, and GCPS-B; (3) PI-max with PCS, IPQ, and GCPS-H; (4) PI-typ with PCS and GCPS-H; and (5) P-ext-B with PHQ4 and PHQstr. Pain duration showed only weak or no correlations with all pain measures. All other axis II measures (IEQ, ISI, and DCQ) only had weak or no correlations with pain features.

From Figure 4B, when correlation analyses were performed after dichotomizing questionnaire scores into above and below cutoff values, we found that PHQstr scores above nine (*N* = 46) significantly correlated with levels of P-ext, PI-typ, ∑-PI-rest-H, and ∑-PI-move-H (*r* = 0.34–0.47; *P* < 0.05). GCPS-B scores above cutoff level (*N* = 107) significantly correlated with P-ext-B and ∑-PI-move-H (*r* = 0.30 and 0.31, respectively; *P* < 0.001). DCQ scores above cutoff level (*N* = 22) moderately correlated (*r* = 0.32–0.41) with P-ext, PI-max, ∑-PI-rest-H, and ∑-PI-move-H. IEQ scores above cutoff level (*N* = 93) significantly correlated with PI-typ (*r* = 0.39; *P* < 0.001). ISI scores above 14 (*N* = 121) significantly correlated with PI-typ (*r* = 0.31; *P* < 0.001).

## Discussion

The aim of this study was to further understand the pain–to–well-being relationship in patients experiencing OFP. Thus, the relationships between various pain features and other psychometric measures as indicators of well-being were analyzed. Furthermore, pain features and psychometric scores were analyzed with regard to patient characteristics, such as gender, age group, employment status, and pain duration.

### Data Origin and Sample Characteristics

Anonymized data were derived from a large clinical cohort of 799 patients who completed the WISE between March 19, 2017 and May 19, 2019. This comprehensive evaluation tool is routinely used in the Orofacial Pain Unit of the Center of Dental Medicine, University of Zurich, to plan the resources for the initial consultation, such as allocation of time and appropriate healthcare specialists. Table 1 indicates that the gender proportion and age distribution matched those reported in other studies on patients seeking care for OFP (16, 38–40). Three quarters of our sample were in training or working, while 6.5% of patients were disabled. Most patients were considered chronic pain sufferers since more than 80% of them experienced pain longer than 3 months (41).

### Pain Scores and Relation to Patient Characteristics

On average, patients reported pain in ~9 of possible 93 locations as defined in the diagrams in Figure 1, indicating that the majority experienced locoregional pain. On the 11-point numerical pain scale, the reported mean pain intensity was moderate (4.9), with a mean maximum intensity of 7.1. As expected, pain was more intense upon movement compared to at rest (Table 2). Pain intensities were higher in women than men and higher in disabled than working patients (Figure 2). As was previously reported, pain is generally more prevalent in females in the head and neck areas (42, 43). Somewhat counterintuitively, pain extension decreased from middle to old age as was reported in fibromyalgia patients (44). This may be due to the observation that OFP origins shift across age from myogenic to neurogenic (e.g., trigeminal neuralgia) and the latter being typically a localized pain (45, 46). This may also explain why working subjects indicated more pain locations than those retired. Recent findings do not indicate that OFP mediates the relationship between socioeconomic inequalities (linked to disability) and the impacts of oral conditions on daily life (47).

### Well-Being Scores

Table 3 summarizes the axis II questionnaire scores serving as proxies for well-being. The PHQ4 is part of the WISE checklist and was therefore completed by all patients. All patients were also asked to complete the PCS due to relevant associations of pain catastrophizing with pain-related disability, number of painful body sites, and pain-related impact (7). The sample prevalence rates of clinically relevant axis II instrument scores (above cutoff) in descending order were as follows: GCPS-H, 27%; PHQ4 and PHQ9, 21%; PCS, 20%; GAD7, 15%; ISI, 15%; IEQ, 14%; GCPS-B, 13%; PHQstr, 6%; and DC, 3%. No cutoff value has been reported for the IPQ. The GCPS can predict the healthcare utilization cost for chronic OFP patients (48). Approximately one in four patients experienced moderate or severe impairment due to craniofacial pain (and 13% due to bodily pain). Almost as many had PHQ scores that warrant further evaluation for depression, whereas 15% experienced symptoms characteristic of a GAD. The 20% of patients reporting pain catastrophizing scores above 29 might have an elevated risk of delayed pain recovery (49). Pain catastrophizing was demonstrated to mediate the effects of distress in OFP patients, which was attributed to the helplessness component of the PCS (50). Due to the sample overlap, the prevalence of moderate and severe grades of insomnia (15%) confirms the 16% we previously reported (13). A novel finding of this study is that approximately one in seven patients (14%) perceived injustice of a clinically relevant level. It is noteworthy that patients concerned about injustices in the treatment they receive are vulnerable to greater emotional distress, prolonged work disability, invalidating or stigmatizing reactions of others, and poor pain-related outcomes (51–53). According to Sullivan (54), blame cognitions may have an impact on feelings of anger and revenge motives that warrant interventions to alter the individual's perceptions of the offender. Although perceived stress has been reported to predict the incidence of painful TMDs, the number of patients (6%) reporting medium to severe stress on the PHQstr was rather low in this sample, likely due to the broad variety of pain origins (16, 18, 29). And only 2% reached clinically relevant DCQ scores. Health anxiety questionnaire was reported to have good association with OFP and other related syndromes (55) and was found to be a strong predictor of chronic OFP (56). However, we only observed moderate correlation between GAD7 score and ∑-PI-rest-H in our subjects. The inclusion of patients experiencing various types of disorders in this study is justified by the pain associations with axis II psychological and psychosocial variables independent of pain origin (57).

### Well-Being Scores in Relation to Patient Characteristics

Women not only experienced significantly more intense pain than men (Figure 2) but also suffered significantly severer impairment (data not shown). A multitude of mechanisms has been proposed to explain sex differences in pain and emotional processing, including the effects of sex hormones, differences in endogenous opioid function, cognitive/affective influences, coping patterns, and contributions of social factors such as stereotypic gender roles (58, 59). In our sample, PHQ4 and PHQstr scores were highest in 50–59-year-old subjects, which is consistent with a Japanese study reporting PHQ scores being low in young adulthood, increasing in middle age (peaking during age 50–59), and then decreasing again in older age (60). The relationship between employment-related factors and chronic pain lacks adequate research (61). Our findings that being disabled and/or unemployed was negatively associated with various axis II instrument scores emphasize the need for routine collection of information on employment status to optimize clinical care and future research (62). It is noteworthy that daily activities are negatively influenced not only by pain itself but also by pain-related fear (63).

### Correlations Between Pain and Well-Being Scores

As the structure of the WISE instrument is modular, not all 799 patients completed each axis II instrument or each pain-related questionnaire. However, everyone was requested to complete questions related to pain locations, intensities, and duration. None of the calculated correlations was strong (*r* ≥ 0.5). The fact that the PCS, PHQ4, and PHQstr were completed by all patients and the GCPS-H by nearly 90% of them largely explains their highest correlation levels with pain intensity variables and pain extension for the entire sample (Figure 4A). Such effects reversed when only cases with clinically relevant scores (above cutoff) were analyzed (Figure 4B). Our data thus support the routine use of PHQ4 as a screener for anxiety and depression in the clinical setting, as recommended by international expert panels (11). PEG, a three-item scale measuring pain intensity (P), interference with enjoyment of life (E), and interference with general activity (G), is another tool available for initial pain patient evaluation (64). Psychological comorbidity has been shown to influence patients' illness perceptions by means of pain ratings, treatment-seeking behavior, and treatment adherence, as well as recovery after surgical procedures (65–67).

The well-being of patients is more likely compromised when we defined compromised well-being by axis II scores at or above cutoff values. Figure 4B reveals that patients experiencing widespread pain and high sum intensity scores suffered high distress levels (PHQstr scores ≥10; N = 46; r = 0.34–0.47; all *P*-values <0.05). This confirms previous findings that self-reported stress independent of its origin (pain or non-pain-related) can initiate or exacerbate the impact of TMDs, chronic pain, and disability (68, 69). However, further research is needed to clarify the amount, timing, severity, and type of stress that is needed to contribute, maintain, and exacerbate persistent pain (70). While stress is often comorbid with anxiety, depression, as well as pain catastrophizing and persistence, clinically relevant scores of these domains did not significantly correlate with any pain measure in our study (6, 71–73). The low number of patients experiencing dysmorphic features (DCQ scores ≥9; *N* = 22) makes it difficult to interpret correlations with pain features. Still, this may be an interesting topic for further exploration.

A new and highly significant finding was that typical pain intensity moderately correlated with high scores on the IEQ (scores >17; *N* = 93). The IEQ has proven to be a good predictor for delayed healing in patients experiencing pain and post-traumatic stress (6, 74, 75). However, we are unaware of the use of this questionnaire in the OFP domain, except for its German-language validation (28). Notably, patients perceiving injustice also had pain for a longer duration compared to subjects with lower IEQ scores (*r* = 0.27; *P* < 0.001).

Although patients with OFP report sleep disturbances disproportionately more often compared with the general population (13), clinically relevant insomnia only associated with typical pain intensity at a moderate level, which is in line with previous observations (ISI scores >15; *N* = 121) (76). This finding indicates that other psychosocial variables likely contribute more to insomnia than the selected pain features, and it supports the notion that sleep impairment is more likely a predictor of OFP than pain is of sleep impairment (77). Surprisingly to us, pain duration did not correlate with any of the axis II measures employed in our study, except for a weak correlation with perceived injustice.

A key difference of this study as compared to the OPPERA studies (78) is that our sample is a pure clinical sample and therefore more likely represents a clinical population that most dentists encounter in their practices. Taken together, our results support Schiffman et al. (11) for the use of GCPS and PHQ4 as screening tools in clinical practice; however, for comprehensive assessments of orofacial patients' well-being, not only PHQ9 and GAD7 but also PHQstr, DCQ, IEQ, and ISI should be utilized by specialists to obtain a more in-depth evaluation of these patients.

Although it was not the main objective of this study, we also explored the correlations between various axis II instruments (data not shown) and, interestingly, found that IPQ and IEQ strongly correlated with PCS and PHQ4 (*r* = 0.55–0.61; *P* < 0.001).

### Study Limitations

Since this is a cross-sectional study, we cannot draw definite causal conclusions regarding the relationship between pain and well-being. The WISE pain drawing allows patients to report on pain intensities in every region marked on the body diagram. When a person experienced pain in multiple locations, the limitation to a single numeric value for the typical (PI-typ) and maximum (PI-max) pain intensity characteristic of his/her chief complaint may have made the choice difficult. We did not attempt to capture psychological factors that influence pain intensity and distribution on pain drawings (79, 80), although we are aware of their importance in understanding the somatic awareness present in chronic pain conditions (81). Since the DC/TMD axis II measures currently do not cover some of the biopsychosocial aspects of interest in this study, which included a broad variety of OFP complaints beyond TMDs, we have incorporated several additional psychometric instruments in our data analyses. Most patients in this study had moderate pain intensities, which may explain why no strong correlation levels were detected in our analyses. The low prevalence of DCQ likely explains the lack of correlation between the DCQ and the assessed pain dimensions. Nevertheless, the fact that the correlation between dysmorphic concern and pain has not previously been reported warrants further investigations. This study did not examine the presence of additional painful comorbidities, although patients experiencing prolonged painful TMDs are at increased risk of suffering from other painful conditions (82). Furthermore, we did not employ a specific instrument for measuring well-being, such as the Warwick–Edinburgh Mental Well-Being Scale (83). Rather, our well-being proxy measures included the set of axis II constructs listed in the Materials and Methods section.

## Conclusions

Due to the modular structure of the WISE that questions patients according to their symptom burden, a variable number of the 799 patients completed the axis II instruments. Pain intensities were higher in women than men and highest in patients with disabilities or no job. The latter group also had the highest scores for pain-related disability, pain catastrophizing, stress, and depression. Older patients (60+ years) had significantly lower stress scores and were less likely to report symptoms of anxiety or depression. When correlating pain and well-being measures, moderate associations were observed in patients reporting clinically relevant stress, insomnia severity, injustice experience, and dysmorphic concern. Surprisingly, compromised well-being in the form of anxiety, depression, and pain catastrophizing (clinically relevant scores of GAD7, PHQ9, and PCS) did not correlate with any of the pain measures. The results of this study support the clinical usefulness of the DC/TMD core assessment instruments, but also suggest measuring of sleep, catastrophizing, injustice experience, and dysmorphic concern as important dimensions for patients suffering from OFP symptoms. Further studies are needed to determine how pain and well-being influence each other, i.e., to clarify whether there are direct causal influences or influences mediated by other variables not investigated in this study. Preferably this would be related to a theoretical framework about relations between OFP, well-being, psychiatric disorders, and socioeconomic status.

## Data Availability Statement

The raw data supporting the conclusions of this article will be made available by the authors, without undue reservation.

## Ethics Statement

Ethical review and approval was not required for the study on human participants in accordance with the local legislation and institutional requirements. Written informed consent to participate in this study was provided by the participants' legal guardian/next of kin.

## Author Contributions

All authors listed have made substantial, direct and intellectual contribution to the work, and approved it for publication.

## Conflict of Interest

The authors declare that the research was conducted in the absence of any commercial or financial relationships that could be construed as a potential conflict of interest.
